# General practice patients treated for substance use problems: a cross-national observational study in Belgium

**DOI:** 10.1186/s12889-016-3885-0

**Published:** 2016-12-08

**Authors:** Nicole Boffin, Jerome Antoine, Sarah Moreels, Simeon Wanyama, Karin De Ridder, Lieve Peremans, Marc Vanmeerbeek, Viviane Van Casteren

**Affiliations:** 1 OD Public Health and Surveillance, Scientific Institute of Public Health, Brussels, Belgium; 2 Département de Médecine Générale, Université de Liège, Liège, Belgium; 3 Department of Primary and Interdisciplinary Care, University of Antwerp, Antwerp, Belgium

**Keywords:** Surveillance systems, Substance abuse, Family practice

## Abstract

**Background:**

General Practitioners (GPs) are well placed to care for patients with (chronic) substance use problems. This pilot was carried out to study the feasibility and usefulness of a continuous surveillance of substance use problems among general practice patients. The objectives were (i) to describe variables with missing values exceeding 1% and whether patients were reported without substance-related problems; (ii) the profile and the magnitude of the patient population that is treated for substance use problems.

**Methods:**

Observational study by the Belgian Network of Sentinel General Practices (SGP) in 2013. Baseline (at the first encounter) and 7-month follow-up data were reported of all patients treated for substance use problems. Two main measurements were type of substance use and patient status at follow-up. Multiple logistic regression analysis was used to examine patient status at follow-up.

**Results:**

Of 479 patients, 47.2% had problems with alcohol alone, 20.3% with prescription drugs, 16.7% with illicit drugs other than heroin or methadone and 15.9% with heroin or methadone. Problems with alcohol alone were more prevalent in Flanders (53.0%; 95% confidence interval (CI) 46.8–59.1%) than in Wallonia-Brussels (39.8%; 95% CI 33.1–46.8%), while problems with heroin or methadone were more prevalent in Wallonia-Brussels (27.0%; 95% CI 21.1–33.5%) than in Flanders (7.1%; 95% CI 4.3–10.9%). At follow-up, 32.8% of the patients had dropped out, 29.0% had discontinued GP treatment and 38.2% had continued GP treatment. Overall, 32.4% of 479 patients had continued GP treatment for substance use problems during the study period. In Wallonia-Brussels, this proportion was higher (42.7%; 95% CI 35.9–49.6%) than in Flanders (24.3%; 95% CI 19.2–29.8%).

**Conclusions:**

A continuous surveillance of the general practice population treated for substance use problems seems to be feasible and useful. The latter is suggested by the specific profile and the relative magnitude of the population. Inter-regional health system differences should be taken into account to estimate the epidemiology of substance use problems among general practice patients.

**Electronic supplementary material:**

The online version of this article (doi:10.1186/s12889-016-3885-0) contains supplementary material, which is available to authorized users.

## Background

Substance abuse is increasingly recognised as a chronic condition that is accompanied by multiple health harms and social problems [1]. The life of many patients with substance use problems is marked by cycles of recovery, relapse, and repeated treatments before reaching stable recovery, permanent disability or death [2]. General practitioners (GPs) are well placed to care for these patients since primary care is essentially first-contact care, long-term person-focused care, comprehensive care for most health problems and coordinated care if care elsewhere is required [3]. The European Study of the Epidemiology of Mental Disorders (ESEMeD) showed that the majority of people who seek care for mental health problems consult a primary caregiver [4]. ESEMeD found that in Belgium 66% of people seeking care for alcohol use disorders consulted a GP and 10% consulted only a GP. Recent research supports the provision of screening and brief interventions for hazardous alcohol use by GPs and substitution treatment for problem drug use [5, 6].

This pilot was carried out to study the usefulness and the feasibility of a continuous surveillance of substance use problems in the general practice population. Baseline and follow-up data from patients treated for substance use problems were reported in 2013 by the Belgian Network of Sentinel General Practices (SGP). Sentinel surveillance is a system of public health surveillance that aims to monitor and clarify the epidemiology of health problems to inform public health policy. In Belgium, 94% of the population has a regular GP and 77% visits their GP annually [7]. Therefore, GPs are considered to be the second best source of public health information after the general population.

There were two reasons for piloting this surveillance. First, it was found that primary care physicians identify fewer than half of patients with alcohol use disorder when using clinical judgement, and that their medical records were accurate in less than three out of ten [8]. In this study, we expect to overcome the problem of non-recognition and thus non-reporting of patients with substance use problems by including only treated patients. Second, general practice is not covered by the Treatment Demand Indicator (TDI) register that was established in 2011 to monitor the epidemiology of substance-related problems in Belgium. The TDI register collects data on new treatment episodes reported by care providers offering treatment for substance-related problems. In contrast to other European TDI registers, treatment by GPs is not included in the Belgian TDI register [9]. Yet, the nature of primary care suggest that general practice patients have less severe problems than those treated in specialised care settings (not setting,) [10]. In the domain of substance use problems, there is some evidence that users of alcohol alone present less severe problems than poly-substance users or drug users [11, 12]. There are also indications that GPs have negative attitudes towards users of illicit drugs [13]. Belgian GPs find the use of alcohol and cannabis more socially acceptable than other drugs [14]. We thus assume that general practice patients treated for substance use problems show a different profile compared to patients treated in specialised care settings. Consequently, the SGP surveillance would be of use, in complement to the TDI register, to estimate the epidemiology of problematic use of substances as reflected by treatment demand.

We also expect a different patient profile in Flanders compared to Wallonia-Brussels due to the organisation of health services. In Wallonia-Brussels the provision of opiate substitution treatment (OST) by GPs is much more common than in Flanders [15, 16].

In short, to pilot (i) the feasibility and (ii) the usefulness of a continuous surveillance study by the SGP the following objectives were set:

(i). To describe variables with missing values exceeding 1% and whether patients were reported without specific substance-related problems. Both can be seen as indicators of data quality. The inclusion of patients without any of the substance-related problems displayed on the registration form conflicts with the concept of “problematic use of substances”.
(ii). To examine the profile and the magnitude of the general practice population that is treated for substance use problems, by their GP alone or mixed with treatment by non-GP caregivers, in Belgium and its regions. Type of substance use is one measure describing the profile of the population. The relative magnitude of the population is described by a second measure, patient status at follow-up, more exactly the proportion of patients who continued substance use treatment by their GP throughout the study period. We examine if type of substance use is a determinant of patient status at follow-up.

## Methods

### Settings and participants

The Belgian Network of SGP comprises approximately 150 general practices with one or more sentinel GPs who purposively record routine clinical care data for the surveillance of specific health problems or care delivery. Data are reported weekly on standard registration forms for a period of at least one year. Sentinel GPs are comparable to non-sentinel GPs for age and gender and the countrywide network covers 1.4 to 1.8% of the Belgian population [17].

This study was carried out on top of the regular 2013 program involving 139 SGP. All SGP were invited to participate in this “Pilot study of patients being treated for problematic use of substances” by using an additional registration form. Pilot participation (*n* = 104 SGP) was not significantly associated with the SGP region, the solo/group character of the SGP, nor the age of participating sentinel GPs (*n* = 181) within all the SGP. The proportion of women sentinel GPs participating in the pilot study (59 of 67, 88.1%) was higher than the proportion of men (77 of 114, 67.5%) (*p* = 0.001).

Baseline data were collected during five months from mid-May to mid-October 2013. Fifty-six percent of the SGP were located in Flanders and 56% of the patients were reported by Flemish SGP. Five-month follow-up forms were sent out for all recorded patients. In February 2014, all 104 SGP who reported at least one patient with substance use problems were sent a list of the reported patients to be returned after correction, completion or confirmation of its content. Due to database administration errors the intended follow-up time was delayed by approximately two months (see results section). Inclusion criteria and definitions were described on the registration form. Sentinel GPs were asked to complete a registration form “at the first contact with all new or regular patients between 18 and 64 years old who received treatment or counselling for problematic use of substances (alcohol, illicit drugs or prescription drugs)”. We included the working age population only as the study was designed in collaboration with the UP TO DATE research consortium investigating the role of occupational health services in substance use problems [18].

We stated that the amount or frequency of substance use was not to be reported as the study was focusing on patients being treated for problematic use of substances. We thus assumed that treatment for substance use was a sufficient indicator of its problematic character and did not further define “problematic use of substances”.

### Variables and measures

Substances displayed on the SGP registration form were alcohol, cannabis, opiates (heroin), methadone (or other substitute), cocaine, ecstasy, psychostimulants (amphetamines), hypnotics, tranquillizers, opioid analgesics, and “other substances”. The measure “type of substance use” was based on a hierarchical classification of substances into four mutually exclusive categories: 1) use of alcohol alone; 2) use of hypnotics/sedatives, tranquillizers or opioid analgesics, with or without alcohol; 3) use of cannabis, stimulants, hallucinogens, cocaine or other illicit drugs but no heroin or methadone, with or without the substances in the two previous categories; and 4) use of heroin or methadone, with or without the previously displayed substances. This classification was based on a literature review and patterns of substance use observed in the SGP data, for example 18 of 25 users of stimulants also used heroin, cocaine, ecstasy or cannabis while only 1 of 31 users of opioid analgesics also did [19]. Although methadone may be prescribed and used as intended by the prescribing physician, it was classified as an illicit drug based on its relation with heroin.

Three substance use characteristics were reported: abstinence from substances in the last four weeks, the length of problematic substance use in years and the occurrence of substance-related problems. The latter were grouped on the standard registration form and in the analysis as physical problems, mental problems, social problems and problems at work. Treatment history at baseline was described by two variables: whether this was a new treatment episode (versus an ongoing) and whether this was the first ever treatment. No criteria were given to define the start and the end of a treatment episode. Types of treatment listed on the registration form were a brief intervention (defined on the form), non-pharmacological or psychological treatment, pharmacological treatment or other treatment.

The measure “patient (treatment) status at follow-up” was defined by five categories based on the occurrence and timing of GP-patient encounters after baseline. Patients without a follow-up report, i.e. having dropped out from the study, were considered together with patients not seen by the GP after baseline (1). In our experience, it is likely that SGP did not return follow-up reports for patients not seen after baseline. Patients were considered as having discontinued the treatment if they were seen after baseline but not in the four weeks preceding the follow-up report (2). Patients seen by the GPs in the four weeks before the follow-up report were considered as having continued substance use treatment by their GP (3–5), although there is no evidence for the validity of the cut-off point of four weeks. Depending on the care setting(s), they were classified as having received non-GP treatment alone (3), mixed treatment, i.e. GP and non-GP treatment (4) or GP treatment alone (5). We thus considered the small number of patients who received usual care from their GP without targeted treatment for substance use problems as having continued GP treatment.

### Statistical analyses

All data are person-based. We used 95% binomial proportion confidence intervals (CI) to describe patient population characteristics and treatment status at follow-up by region. Significant bivariate associations were examined by multiple logistic regression. We used multiple logistic regression analysis to evaluate whether type of substance use was a determinant of 1) continued treatment for substance use problems by the GP alone and 2) a mix of GP and non-GP treatment. Multinomial analysis of five categories of treatment status at follow-up was excluded due to small cell numbers. All patient population characteristics that were significantly (*p* < 0.05) associated with the dependent variables were included in both the full models except work status and work problems in order to avoid overadjustment bias [20]. A generalised estimating equation approach was used to account for the clustering of data within general practices. All possible interaction effects between independent variables were tested. Data were analysed with Stata 13.

## Results

### Study data flow

**Fig. 1:**
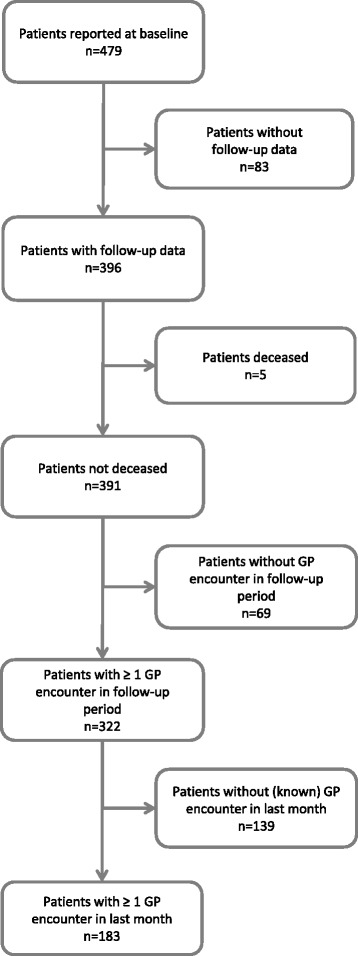
Study data flowᅟ

At baseline 479 general practice patients receiving treatment for substance use problems were recorded (Fig. 1. Study data flow). No follow-up data were reported for 83 patients, resulting in a study drop-out of 17.3%. Study drop-out was not significantly associated with any of the baseline patient characteristics or with the SGP region (data not shown). A median follow-up reporting delay of 66 days (interquartile range (IQ) 47–77) was observed, resulting in a median follow-up time of 7 months. There was no significant association between delay of follow-up and treatment status at follow-up (data not shown).

### Missing data and inclusion of patients without specific substance-related problems

Missing data exceeding 1% were observed for the length of problematic substance use in years (22.1%), whether this was the first treatment episode (8.8%), occupational status (4.4%) and whether this was a new treatment episode (3.1%) (Table 1).

**Table 1:** Patient population characteristics by region (*N* = 479)

	Flanders (*N* = 268)		Wallonia & Brussels (*N* = 211)		Belgium (*N* = 479)	
	n/N	% (95% CI)	n/N	% (95% CI)	n/N	% (95% CI)
Men (versus women)	177/267	66.3 (60.3–71.9)	138/207	66.7 (59.8–73.0)	315/474	66.5 (62.0–70.7)
Age in years						
<25	16/268	6.0 (3.5–9.5)	8/211	3.8 (1.7–7.3)	24/479	5.0 (3.2–7.4)
25–34	40/268	14.9 (10.9–19.8)	42/211	19.9 (14.7–25.9)	82/479	17.1 (13.9–20.8)
35–44	50/268	18.7 (14.2–23.8)	46/211	21.8 (16.4–28.0)	96/479	20.0 (16.5–23.9)
45–54	102/268	38.1 (32.2–44.2)	64/211	30.3 (24.2–37.0)	166/479	34.7 (30.4–39.1)
55–64	60/268	22.4 (17.5–27.9)	51/211	24.2 (18.6–30.5)	111/479	23.2 (19.5–27.2)
Type of substance use						
Alcohol alone	142/268	**53.0 (46.8–59.1)**	84/211	**39.8 (33.1–46.8)**	226/479	47.2 (42.6–51.8)
Prescription drugs	53/268	19.4 (14.8–24.7)	44/211	20.9 (15.6–27.0)	97/479	20.3 (16.5–23.9)
Illicit drugs, excluding heroin and methadone	54/268	20.1 (15.5–25.5)	26/211	12.3 (8.2–17.5)	80/479	16.7 (13.5–20.3)
Heroin or methadone	19/268	**7.1 (4.3–10.9)**	57/211	**27.0 (21.1–33.5)**	76/479	15.9 (12.7–19.5)
Substance use characteristics						
Abstinence from substances in last 4 weeks	108/268	**40.3 (34.4–46.4)**	118/211	**55.9 (48.9–62.7)**	226/479	47.2 (42.6–51.8)
Single substance use	178/268	66.4 (60.4–72.0)	127/211	60.2 (53.2–61.8)	305/479	63.7 (59.2–68.0)
Problematic use ≥10 years	102/209	**48.8 (41.8–56.0)**	111/164	**67.9 (59.9–74.8)**	213/373	57.1 (51.9–62.2)
Substance-related problems						
Physical problems	149/268	**55.6 (49.4–61.6)**	84/211	**39.8 (33.2–46.8)**	233/479	48.6 (44.1–53.2)
Mental problems	206/268	76.9 (71.3–81.8)	172/211	81.5 (75.6–86.5)	378/479	78.9 (75.0–82.5)
Problems at work	61/268	22.8 (17.9–28.3)	30/211	14.2 (9.8–19.7)	91/479	19.0 (15.6–22.8)
Social problems	198/268	73.9 (68.1–79.0)	153/211	72.5 (59.0–78.4)	351/479	73.3 (69.1–77.2)
Occupational status: at work	114/253	45.1 (38.8–51.4)	66/205	32.2 (25.9–39.1)	180/458	39.3 (34.8–43.9)
Treatment history						
First treatment episode	66/249	26.5 (21.1–32.4)	39/188	20.7 (15.2–27.2)	105/437	24.0 (20.1–28.3)
Ongoing treatment episode	154/254	60.6 (54.3–66.7)	150/210	71.4 (64.8–77.4)	304/464	65.5 (61.0–69.8)
Treatment status at follow-up						
1) Study drop-out, treatment drop-out or deceased^a^	93/268	34.7 (29.0–40.7)	64/211	30.3 (24.2–37.0)	157/479	32.8 (28.6–37.2)
2) Discontinued GP treatment	91/268	34.0 (28.3–40.0)	48/211	22.7 (17.3–29.0)	139/479	29.0 (25.0–33.3)
3) Continued (usual) GP treatment, substance use treatment by non-GP	19/268	7.1 (4.3–10.9)	9/211	4.3 (2.0–7.9)	28/479	5.8 (3.9–8.3)
4) Continued substance use treatment by GP & non-GP	33/268	12.3 (8.6–16.9)	40/211	19.0 (13.9–24.9)	73/479	15.2 (12.1–18.8)
5) Continued substance use treatment by GP alone	32/268	**11.9 (8.3–16.4)**	50/211	**23.7 (18.1–30.0)**	82/479	17.1 (13.9–20.8)
Continued GP treatment (total of 3 to 5)	84/268	**31.3 (25.8–37.3)**	99/211	**46.9 (40.0–53.9)**	183/479	38.2 (33.8–42.7)
Continued GP substance use treatment (total of 4 and 5)	65/268	**24.3 (19.2–29.8)**	90/211	**42.7 (35.9–49.6)**	155/479	32.4 (28.2–36.8)

*CI* Confidence interval. (Borderline) non-overlapping confidence intervals are in bold

^a^5 of 479 patients were deceased, 2 deaths were caused by substance use

Missing data: gender: *n* = 5 (1.0%); age: *n* = 0; substances used: *n* = 0; length of use: *n* = 106 (22.1%); occupational status: *n* = 21 (4.4%); first treatment: *n* = 42 (8.8%); ongoing treatment episode: *n* = 15 (3.1%)

No single substance-related problem was reported from 13 patients (2.7%).

### Type of substance use and related characteristics

Significant regional differences were found for type of substance use, abstinence from substances in the four weeks preceding the baseline encounter, length of problem use, occurrence of physical substance-related problems and treatment status at follow-up (Table 1). Problems of alcohol alone were significantly more prevalent in Flanders compared to Wallonia-Brussels, where problems of heroin or methadone were much more important (see Additional file 1: Table S1).

One hundred and ten of 233 (47%) patients with physical problems had gastro-intestinal problems (Table 1). The odds of physical problems were significantly higher among patients in Flanders (odds ratio (OR) 1.66; 95% CI 1.01–2.73), even after adjustment for type of substance use (data not in table). The odds of a long history of substance use problems (≥10 years) were borderline significantly higher for patients in Wallonia-Brussels (OR 1.75; 95% CI 1.00–3.10) after adjustment for type of substance use (data not in table). The odds of recent abstinence remained significantly higher in Wallonia-Brussels (OR 2.01; 95% CI 1.42–3.10), independent of type of substance use (data not in table).

### Patient status at follow-up

The total drop-out rate of 32.8% (157 of 479 patients) includes study drop-out (*n* = 83), treatment drop-out (*n* = 69) and patients’ death (*n* = 5) (Fig. 1 and Table 1). The treatment discontinuation rate was 29%, in other words, 139 of 479 patients had at least one GP encounter during the study period but not in the four weeks before the follow-up report. The total treatment continuation rate of 38.2% means that 183 of 479 patients continued GP substance use treatment during the study period, i.e. they had a GP encounter in the four weeks before follow-up reporting. The smallest part of the GP treatment continuation group (28 patients, 5.8%) received treatment as usual by their GP and substance use treatment by a specialised caregiver. Seventy-three patients (15.2%) received substance use treatment by their GP and a non-GP caregiver and 82 patients (17.1%) received substance use treatment by their GP alone.

Continued substance use treatment by a GP, alone or mixed with non-GP treatment, was higher among users of heroin or methadone, patients with an ongoing treatment episode and patients in Wallonia-Brussels (Table 2). The adjusted model revealed that type of substance use is not an independent determinant of continued substance use treatment by a GP (alone or mixed), only the region is (Table 3).

**Table 2:** Rates of continued GP substance use treatment, respectively by the GP alone or mixed with non-GP treatment, and by the GP alone, according to patient characteristics

	Continued substance use treatment by GP (alone or mixed with non-GP)				Continued substance use treatment by GP alone			
	Yes (*n* = 155)		No (*n* = 324)^a^		Yes (*n* = 82)		No (*n* = 397)^b^	
	n/N	% (95% CI)	n/N	% (95% CI)	n/N	% (95% CI)	n/N	% (95% CI)
Men (versus women)	96/154	62.3 (54.2–70.0)	219/320	68.4 (63.0–73.5)	55/82	67.0 (55.8–77.1)	260/392	66.3 (61.4–71.0)
Age in years								
<25	7/155	4.5 (1.8–9.1)	17/324	5.2 (3.1–8.3)	1/82	1.2 (0.3–6.6)	23/397	5.8 (3.7–8.6)
25–34	22/155	14.2 (9.1–20.7)	60/324	18.5 (14.4–23.2)	4/82	**4.9 (1.3–12.0)**	**78/397**	**19.6 (15.9–23.9)**
35–44	34/155	21.9 (15.7–29.3)	62/324	19.1 (15.0–23.8)	20/82	24.4 (15.6–35.1)	76/397	19.6 (15.9–23.9)
45–54	61/155	39.4 (31.6–47.5)	105/324	32.4 (27.3–37.8)	39/82	47.6 (36.4–58.9)	127/397	32.0 (27.4–36.8)
55–64	31/155	20.0 (14.0–27.2)	80/324	24.7 (20.1–29.8)	18/82	22.0 (13.6–32.5)	93/397	23.4 (19.3–27.9)
Type of substance use								
Alcohol alone	64/155	41.3 (33.5–49.5)	162/324	50.0 (44.4–55.6)	33/82	40.2 (9.6–51.7)	193/397	48.6 (43.6–53.7)
Prescription drugs	36/155	23.2 (16.8–30.7)	61/324	18.5 (14.4–23.2)	25/82	30.5 (20.8–41.6)	72/397	17.9 (14.2–22.0)
Illicit drugs, excl. heroin and methadone	18/155	11.6 (7.0–17.7)	62/324	19.1 (15.0–23.8)	8/82	9.8 (4.3–18.3)	72/397	18.1 (14.5–22.3)
Heroin or methadone	37/155	**23.9 (17.4–31.4)**	39/324	**12.0 (8.7–16.1)**	16/82	19.5 (11.6–29.7)	60/397	15.1 (11.7–19.0)
Substance use characteristics								
Abstinence in last 4 weeks	76/155	49.0 (40.9–57.2)	150/324	46.3 (40.8–51.9)	44/82	53.7 (42.3–64.7)	182/397	45.8 (40.9–50.9)
Single substance use	94/155	60.6 (52.5–68.4)	211/324	65.1 (59.7–70.3)	58/82	70.7 (59.6–80.3)	247/397	62.2 (57.2–67.0)
Problematic use ≥10 years	70/155	57.9 (48.5–66.8)	143/324	56.7 (50.4–62.9)	36/82	56.3 (43.3–68.6)	177/309	57.3 (51.6–62.9)
Substance use related problems								
Physical problems	71/155	45.8 (37.8–54.0)	162/324	50.0 (44.4–55.6)	37/82	45.1 (34.1–56.5)	196/397	49.4 (44.3–54.4)
Mental problems	129/155	83.2 (76.4–88.7)	249/324	76.9 (71.9–81.3)	68/82	82.9 (73.0–90.3)	310/397	78.1 (73.7–82.1)
Problems at work	26/155	16.8 (11.3–23.6)	65/324	20.1 (15.8–24.8)	8/82	9.8 (4.3–18.3)	83/397	20.9 (17.0–25.2)
Social problems	118/155	76.1 (68.6–82.6)	233/324	71.9 (66.7–76.7)	62/82	75.6 (64.9–84.4)	289/397	72.9 (68.1–77.1)
Occupational status: at work	47/155	31.5 (24.2–39.7)	133/309	43.0 (37.4–48.8)	27/82	34.6 (24.2–46.2)	153/380	40.3 (35.3–45.4)
Treatment history								
First treatment episode	32/155	21.6 (15.3–29.1)	73/289	25.3 (20.4–30.7)	22/82	27.2 (18.3–39.1)	83/358	23.2 (18.9–27.9)
Ongoing treatment episode (versus new)	39/155	**74.8 (67.2–81.5)**	188/309	**60.8 (55.2–66.3)**	20/82	75.6 (64.9–84.4)	242/382	63.4 (58.3–68.2)
Wallonia-Brussels (versus Flanders)	90/155	**58.1 (49.9–65.9)**	121/324	**37.3 (32.1–42.9)**	50/82	**61.0 (49.6–71.6)**	161/397	**40.6 (35.7–45.6)**

*CI* Confidence interval. Non-overlapping confidence intervals are in bold

^a^This subpopulation includes all other patients, i.e. dropped-out or deceased patients, patients who discontinued GP treatment and patients who continued usual GP treatment without GP substance use treatment

^b^This subpopulation includes all other patients, i.e. dropped-out or deceased patients, patients who discontinued GP treatment, and patients who continued GP treatment combined with non-GP treatment

**Table 3:** Adjusted odds ratios for continued substance use treatment by the GP alone or mixed with non-GP treatment (*n* = 459)

	OR (95% CI)
Median age or older (versus < median)	1.03 (0.62–1.71)
Men (versus women)	0.79 (0.49–1.29)
Type of substance use	
Alcohol alone	reference
Prescription drugs	1.40 (0.81–2.42)
Illicit drugs, excluding heroin and methadone	0.85 (0.47–1.51)
Heroin or methadone	1.59 (0.80–3.16)
Ongoing treatment episode (versus new)	1.57 (0.97–2.53)
Wallonia-Brussels (versus Flanders)	**1.97 (1.16–3.34)**

*OR* Odds ratio, *CI* Confidence interval. Non-overlapping confidence intervals are in bold

Continued substance use treatment by a GP alone was higher among patients between 45 and 54 years, lower among patients aged 25 to 34 years and equally higher in Wallonia-Brussels (Table 2).

The adjusted model showed an interaction between type of substance use and region. In the Flemish population, the odds for continued substance use treatment by the GP alone were higher for patients with problematic use of prescription drugs and patients with problems of heroin or methadone (Table 4). No significant model of determinants of continued substance use treatment by the GP alone could be developed for the Walloon-Brussels population.

**Table 4:** Adjusted odds ratios for continued substance use treatment by the GP alone in Flanders^a^ (*n* = 267)

	OR (95% CI)
Median age or older (versus < median)	2.44 (0.96–6.25)
Men (versus women)	0.82 (0.36–1.87)
Type of substance use	
Alcohol alone	reference
Prescription drugs	**4.04 (1.65–9.94)**
Illicit drugs, excluding heroin and methadone	0.78 (0.15–4.10)
Heroin or methadone	**6.64 (1.76–25.06)**

*OR* Odds ratio, *CI* Confidence interval. Non-overlapping confidence intervals are in bold

^a^In the overall model of determinants of GP treatment alone, there is a significant interaction between region and type of substance use. Modeling determinants in populations split by region resulted in a non-significant model in Wallonia-Brussels (*n* = 207)

## Discussion

### Main findings

This pilot shows positive results regarding the feasibility of a continuous surveillance of treated substance use problems by the SGP. Few variables had more than 1% missing values. The negligible percentage of patients from whom no substance-related problems were reported suggests a clear understanding of the concept of “problematic use of substances”. The usefulness of a continuous surveillance is suggested by the profile and magnitude of the population. The Belgian general practice population that is treated for substance use problems is characterized by problems of mono-substance use, mostly of alcohol alone. One third of the patients received continued substance use treatment by their GP during the study period, be it by their GP alone or by their GP and a non-GP caregiver. The different profile of general practice patients compared to patients treated in specialised care settings was confirmed. As described in the introduction, general practice patients, known to be relatively old and female, have less severe problems. Problems of alcohol alone are less complex than poly-drug use, according to experiences of GPs and according to patient-related and substance-related characteristics. Half of the population had these problems for 10 years or more and for three quarter of the patients this was not the first treatment. The latter two findings confirm the chronic character of substance use problems in the general practice population.

As expected, we found important regional differences. In Flanders, more than half of the population had problems of alcohol alone while problems of heroin or methadone were more prevalent in Wallonia-Brussels. Treatment continuation was significantly higher in Wallonia-Brussels compared to Flanders. Given its higher level of urbanization, Flanders counts more specialised centres and pharmacies providing OST than Wallonia. A previous study confirmed that OST is mostly supplied by specific, low threshold services in Flanders, while it is mainly offered by GPs in the French community [21]. Although problems of alcohol alone were more prevalent among patients in Flanders (53.0%), their prevalence was similarly considerable in Wallonia-Brussels (39.8%).

### Strengths and weaknesses

As far as we know this is the first nationwide study in Belgium assessing the profile and magnitude of the adult general practice population with treated substance use problems. It is based on usual care data reported by members of a long-standing, multi-subject and representative network of SGP. Provider-reported data are commonly used in epidemiological research on substances use. They offer information on hidden and socially stigmatised populations and can be done on rather low budgets [22]. This study includes a reasonable sample size and has a relatively low and unbiased rate of dropouts. We succeeded in overcoming the problem of under-recognition and under-reporting of substance use problems by GPs by focussing on treated, thus acknowledged, problems. We demonstrated the importance of confounder control at the health system level when using treatment-based data to estimate the epidemiology of problems.

This study has several weaknesses. Our study does not include people with untreated substance use problems, and many people with alcohol problems do not seek treatment [23]. Instructions and definitions presented to the SGP participants were brief since the annual surveillance program comprises multiple health problems. One example is that we did not define when a treatment episode starts or ends. The two main measures, type of substance use and patient (treatment) status at follow-up, lack detailed information. We do not know if methadone was misused or used as prescribed by the GP, and if this prescription was intended for intoxication or for maintenance. Almost all patients were found to have at least one substance-related problem but the clinical diversity of the population is large. Yet, not only do patients with substance use problems form a heterogeneous group, so do their GPs and their interventions. Moreover, facts and views on problematic substance use are inseparable from the care setting. Problem use of tranquillizers and sedatives is undoubtedly much more common among general practice patients than is the non-medical use or misuse of prescription drugs by illicit drug users, e.g. heroin users. These aforementioned weaknesses are largely due to the use of provider-reported, care-based data. Another set of weaknesses is related to the pilot character of the study. The short study period relative to the large number of measures resulted in insufficient statistical power to apply optimal analysis such as multinomial analysis of all categories of patient status at follow-up. A second example is that we were unable to adjust for individual substances in the model exploring determinants of continued substance use treatment by a GP. The data were collected only by part of the SGP network and over a limited time period. The study sample may be biased towards SGP with relatively more experience with substance use problems. We may have missed patients treated in a residential setting and thus unable to visit their GP in the short study period. In future, this potential bias may be overcome by asking SGP to report any follow-up information about the patient, including information from other caregivers.

### Comparison with evidence (from literature)

It would make little sense to compare our findings with the 2012 TDI report describing new treatment episodes only from the treatment centres under contract with the National Institute for Health and Disability Insurance (NIHDI) [24]. Neither is it easy to compare our findings with findings from the general population. The Belgian Health Interview Survey 2013 does not allow regional comparisons because of the relatively low use of (illicit) drugs in the general population [7]. It shows no regional differences in problematic alcohol consumption by people aged 18–64 years, according to three indicators of alcohol consumption (lifetime problematic drinking based on CAGE 2+, daily drinkers with hazardous drinking habit and weekly consumption of 6+ drinks per occasion). However, alcohol-related premature mortality exhibits a clear-cut regional divide with higher rates in Wallonia and Brussels and lower rates in Flanders [25].

For some study findings we did not find a straightforward explanation. In Wallonia-Brussels, fewer patients suffered from substance-related physical problems despite a longer history of use. Recent abstinence of substances remained higher in the latter region, independent of type of substance use.

### Implications

Our findings suggest that general practice should be taken into account to provide an indicator of treatment demand since 17% of the SGP population was treated by their GP alone during the study period. Yet, the SGP study period was less than one year and eventually all general practice patients from our sample may have started a new treatment episode in a non-general practice setting in the course of 2013. The relatively large proportion of missing values for the length of use in years may be diminished by grouping years in categories on the registration form. The Belgian TDI register equally shows a rather large proportion of missing values for the variable first treatment [24]. Ideally, a continuous surveillance by the network of SGP should follow the TDI protocol as closely as possible without overloading busy GPs with instructions. In the near future, it will be theoretically possible to measure overlap between the two databases (SGP and TDI) at the patient level by using unique patient identifiers. The organisation of data exchange in the Belgian health care setting according to the principle of a single multifunctional data collection is now ongoing through the implementation of the E-health roadmap (http://www.rtreh.be/EHEALTH/_images/20130419actieplan_egezondheidnl.pdf).

## Conclusions

A continuous surveillance of the general practice population treated for substance use problems seems to be feasible and useful. The usefulness is evidenced by the profile and the relative magnitude of the population. Both study measures show large inter-regional differences that are likely to be associated with differences in health services organisation. These differences in treatment delivery should be taken into account when estimating the epidemiology of substance use problems among general practice patients.

## Additional file

Additional file 1: Table S1. Individual substances used by region (N=479). (DOCX 17 kb)

## Abbreviations

CI: Confidence interval
ESEMeD: European Study of the Epidemiology of Mental Disorders
GP: General practitioner
IQ: Interquartile range
OR: Odds ratio
OST: Opiate substitution treatment
SGP: Sentinel General Practices
TDI: Treatment Demand Indicator
