# Supplementary material for: General practice patients treated for substance use problems: a cross-national observational study in Belgium
Source: BMC Public Health. 2016 Dec 8;16:1235. doi: 10.1186/s12889-016-3885-0 (PMC5143443; doi:10.1186/s12889-016-3885-0)
Supplement: Additional file 1: Table S1. — Individual substances used by region (N=479). (DOCX 17 kb) [file 12889_2016_3885_MOESM1_ESM.docx]

Supplementary Table 1. Individual substances used by region (N=479)

|  | Flanders (n=268) | | Wallonia & Brussels (n=211) | |
| --- | --- | --- | --- | --- |
|  | n | %(95%CI) | n | %(95%CI) |
| Alcohol | 208 | **77.6(72.1-82.5)** | 128 | **60.7(53.7-67.3)** |
| Cannabis | 52 | 19.4(14.8-24.7) | 50 | 23.7(18.1-30.0) |
| Heroin | 18 | **6.7(4.0-10.4)** | 43 | **20.4(15.2-26.5)** |
| Methadone | 4 | **1.5(0.4-3.8)** | 49 | **23.2(17.8-29.5)** |
| Opioid analgesics | 16 | 6.0(3.4-9.5) | 15 | 7.1(4.0-11.5) |
| Any opiates | 35 | **13.1(9.3-17.7)** | 72 | **34.1(27.8-40.9)** |
| Cocaine | 22 | 8.2(5.2-12.2) | 17 | 8.1(4.8-12.6) |
| Ecstasy | 6 | 2.2(0.1-4.8) | 3 | 1.4(0.3-4.1) |
| Amphetamines | 13 | 4.9(2.6-8.2) | 5 | 2.4(0.8-5.4) |
| Stimulants (ecstasy or amphetamines) | 18 | 6.7(4.0-10.4) | 7 | 3.3(1.3-6.7) |
| Hypnotics/sedatives or tranquillizers | 55 | 20.5(15.9-25.9 | 42 | 19.9(14.7-25.9) |
| *Note*: CI Confidence interval. Non-overlapping confidence intervals are in **bold**. | | | | |
